# The Correlates of Body Composition with Heart Rate Recovery after Step Test: An Exploratory Study of Malaysian Adolescents

**DOI:** 10.1371/journal.pone.0082893

**Published:** 2013-12-05

**Authors:** Redzal Abu Hanifah, Mohd. Nahar Azmi Mohamed, Zulkarnain Jaafar, Nabilla Al-Sadat Abdul Mohsein, Muhammad Yazid Jalaludin, Hazreen Abdul Majid, Liam Murray, Marie Cantwell, Tin Tin Su

**Affiliations:** 1 Sports Medicine Unit, Faculty of Medicine, University Malaya, Kuala Lumpur, Malaysia; 2 Centre for Population Health (CePH), Department of Social and Preventive Medicine, Faculty of Medicine, University Malaya, Kuala Lumpur, Malaysia; 3 Department of Paediatrics, Faculty of Medicine, University Malaya, Kuala Lumpur, Malaysia; 4 Centre for Public Health, School of Medicine, Dentistry and Biomedical Science, Queen’s University, Belfast, Northern Ireland; Washington Hospital Center, United States of America

## Abstract

**Background:**

In adults, heart rate recovery is a predictor of mortality, while in adolescents it is associated with cardio-metabolic risk factors. The aim of this study was to examine the relationship between body composition measures and heart rate recovery (HRR) after step test in Malaysian secondary school students.

**Methods:**

In the Malaysian Health and Adolescents Longitudinal Research Team (MyHEART) study, 1071 healthy secondary school students, aged 13 years old, participated in the step test. Parameters for body composition measures were body mass index z-score, body fat percentage, waist circumference, and waist height ratio. The step test was conducted by using a modified Harvard step test. Heart rate recovery of 1 minute (HRR1min) and heart rate recovery of 2 minutes (HRR2min) were calculated by the difference between the peak pulse rate during exercise and the resting pulse rate at 1 and 2 minutes, respectively. Analysis was done separately based on gender. Pearson correlation analysis was used to determine the association between the HRR parameters with body composition measures, while multiple regression analysis was used to determine which body composition measures was the strongest predictor for HRR.

**Results:**

For both gender groups, all body composition measures were inversely correlated with HRR1min. In girls, all body composition measures were inversely correlated with HRR2min, while in boys all body composition measures, except BMI z-score, were associated with HRR2min. In multiple regression, only waist circumference was inversely associated with HRR2min (p=0.024) in boys, while in girls it was body fat percentage for HRR2min (p=0.008).

**Conclusion:**

There was an inverse association between body composition measurements and HRR among apparently healthy adolescents. Therefore, it is important to identify cardio-metabolic risk factors in adolescent as an early prevention of consequent adulthood morbidity. This reiterates the importance of healthy living which should start from young.

## Introduction

Heart rate recovery (HRR) is the rate where the heart beat declines to resting levels after an exercise is performed [1]. HRR is mediated by the autonomic nervous system (ANS), with the rate in the early phase being controlled by the parasympathetic reactivation and later by the withdrawal of sympathetic activity [2-5]. HRR is considered a predictor of cardiovascular related mortality and all-cause mortality in all healthy adult patients. A decreased parasympathetic activity is identified to increase the risk [6-8]. In healthy children, HRR is associated with cardio-metabolic risk factors [9-11]. HRR manifests faster in children than in adults [12], with the rate declining faster in boys than in girls [13]. It becomes slower as children progress in age. However, regular physical activities and exercises can blunt the age effect [14].

The prevalence and health consequences of obesity are a growing pandemic in the world, including Malaysia [15-18]. Childhood obesity, in particular, has been shown to be associated with a compromised ANS control of the heart [19,20]. In children, the measurement of obesity includes looking at body mass index (BMI), waist circumference (WC), waist to height ratio (WHtR), body fat percentage and the sum of skinfold thickness. However, the relationship and predictive value of these body composition parameters to various cardio-metabolic risk factors is still unclear [21,22]. Several studies on paediatric population showed that BMI is the best predictor for cardio-metabolic risk factors [23-27]. However, BMI does not differentiate fat mass from fat free mass in thinner children, but in relatively fat children BMI is a validated index of adiposity[28]. Thus, it is suggested that WC and WHtR are better predictors [29]. WC is an indicator for intra-abdominal obesity in children and adolescent [30] and it is strongly associated with cardio-metabolic risk factors [31-33]. WHtR is also considered as an indicator of intra-abdominal obesity [34,35]. Compared to WC, WHtR is not influenced by age and gender in children [36], and it is also a good predictor of cardio-metabolic risks [37-39]. Body fat percentage recorded from non-invasive bioelectrical impedance analyser (BIA) is also associated with increased risk [40,41]. Some studies suggested that BIA is an accurate predictor of body composition in both adolescent and adults [42-44]. Interestingly there are also studies that indicated BMI and WC as equal predictors of cardio-metabolic risk factors [21,25].

Previous studies have investigated the association of HRR with a cluster of cardio-metabolic risk factors in children and adolescents [9-11]. One study showed that age, gender, pulse rate, and BMI accounted for 39% of the variance to HRR [9]. Another study indicated that WC was the only predictor that was associated with HRR in boys, whereas for girls the predictors include systolic blood pressure, serum glucose and serum C reactive protein. A more current study showed that diastolic blood pressure was inversely associated in girls but in boys it was systolic blood pressure, homeostasis model assessment, WC and skinfold thickness [11]. Looking at these results, body composition parameters appear to be consistently associated with HRR.

Thus far, there are no studies investigating other types of body composition such as WHtR and body fat percentage, or even tests to determine which body composition parameters has the strongest predictive value for HRR in adolescents. This gap is of interest since obesity in adolescents is associated with a compromised ANS control of the heart [19,20]. Moreover, obesity frequently continues into adulthood, and consequently, can lead to health complications such as cardiovascular and metabolic diseases [45] in adults. In this regard, locating a suitable body composition parameter which has the strongest predictive value for HRR is of clinical interest for mass screening purposes and subsequent medical management. 

Previous studies involved with exercise testing were conducted in laboratory settings, using either the treadmill or cycle ergometer protocol [9-14]. The limitations of laboratory based studies include the procurement of specialised equipment and technicians, and longer time period to complete the study. Laboratory settings also make it difficult to extrapolate the results to population-based studies. 

The aim of this study is twofold: 1) to determine the association of HRR with BMI, WC, WHtR , and body fat percentage; and 2) to identify which body composition parameter in the mass screening of Malaysian adolescents, can be considered as the strongest predictor of HRR after step test. 

## Materials and Methods

### Ethics Statement

Ethical approval which complied with the International Conference on Harmonization - Guidelines for Good Clinical Practice (ICH-GCP) and the Declaration of Helsinki, was obtained from the Medical Ethics Committee of the University of Malaya Medical Centre, Malaysia (IRB number 896.34).

### Study Design

The Malaysian Health and Adolescents Longitudinal Research Team (MyHEART) is an on-going prospective longitudinal cohort study that involves three states in the northern and central zone of Malaysia – Perak, Selangor and Kuala Lumpur. The objective of the study is to identify the trends of prevalence of non-communicable diseases’ risk factors among adolescents in Peninsular Malaysia, and to determine how lifestyles in early adolescence influence the development of chronic non-communicable disease in early adulthood. 

Participants of this study comprise students of both genders who are studying in government secondary schools and who read and understand the national language, *Malay*. Participants in boarding, religious, or vernacular schools were excluded as they are not representative of Malaysian schools where majority students attend. In Malaysia, secondary school starts from ages of 13 to 17 years old.

Using the formula *n=* (*z* ^*2*^ *. p.q/r.e* ^*2*^)*x Design Effect*, and the prevalence of smoking among 13 to 15 year olds in school based adolescents in Malaysia as 33% [46], the total sample size calculated for this study was at 1500. 

The total number of secondary schools in the three states involved in this study was 595. The method of sampling and randomization was based on two stages of cluster sampling. The schools were primary sampling units and the students were secondary sampling units. Based on a feasibility study done in 2011 that had response rate of 50% (80-120 students per school), 15 schools were randomly selected as a cluster from a computer generated random number list. Eight schools were from the urban area, and seven schools from the rural area. All Form One (13 years old) adolescents in these schools were invited to participate in the study. All were presented with consent forms for their parents, agreement forms for themselves, and information sheets detailing the study. Participants were those who attended schools during the study and who had handed in both the agreed written consent forms of their parents and individual agreement forms. The MyHEART study lasted three months, from March to May 2012. 

### Baseline and Anthropometric Data

First the participant’s socio-demographic data such as date of birth, age, and gender were collected. Participants were then asked to fill in a standardised form under the supervision of trained enumerators. Once the forms were completed, their blood pressures and pulse rates were measured by medically trained persons who were either paediatricians, medical officers or staff nurses. The participants sit upright with his or her right upper arm positioned at the level of the heart with both feet flat on the floor. They were allowed to relax for 5 minutes before their blood pressure and pulse rate were taken. Both their systolic (SBP) and diastolic blood pressure (DBP) were obtained using a stethoscope and a mercurial sphygmomanometer (CK-101C, Spirit Medical Co., Taiwan). Three readings of blood pressure were taken with 2 minutes interval between each reading. The mean SBP and DBP were used in the analysis. Their pulse rate was taken using a finger pulse oximeter (Baseline 12-1926 Fingertip Pulse Oximeter, Fabrication Enterprises Inc., USA).

Height was taken without socks and shoes with a calibrated vertical stadiometer (Seca Portable 217, Seca, UK) and was recorded to the nearest 0.1cm. Focusing on light clothings, weight was measured with a digital electronic weighing scale (Seca 813, Seca, UK) and was recorded to the nearest 0.1 kilogram. BMI was calculated by using weight in kilograms divided by the square of height in meters. BMI z-score for age and gender was calculated using the World Health Organisation (WHO) Anthro Software version 3.2.2 for SPSS macro, based on WHO reference 2007 (WHO,Geneva, Switzerland). Body fat percentage was measured using a portable body composition analyzer (Tanita SC 240 MA Portable Body Composition Analyser, Tanita Europe B.V., The Netherlands). Particapants’ WC was then measured with a non-elastic measuring tape (Seca 201, Seca, UK) that is positioned mid-way between the lowest rib margin and the iliac crest. Measurement was calculated to the nearest millimetre. WHtR was calculated by considering WC in cm divided by height in cm.

### Exercise Test

The exercise test was performed under the close supervision of a sports physician. All participants were first screened before the actual testing. Participants with known medical conditions, musculoskeletal injuries, or who were acutely ill were excluded. The modified Harvard Step Test protocol was chosen as a tool for exercise test because it is one of the step tests developed that objectively categorise the performance level of children. This tool has been successfully used by others among this age group where a 30 cm high step box was applied [47-49]. The 30cm high step box was a typical step box used in Malaysian schools for fitness assessment. The process began with the participants got onto the step box and off the step box at a pace of 30 cycles per minute with a metronome set at 120 beats per minute (bpm), for a total of 5 minutes. A finger pulse oximeter (Baseline 12-1926 Fingertip Pulse Oximeter, Fabrication Enterprises Inc., USA) was attached to one of the student’s fingers, and the pulse rate was then continuously monitored. The peak pulse rate of each student during each minute of the step box exercise was recorded. Those with a pulse rate of 200 bpm, and those who had difficulty in breathing, or were unable to finish, were stopped immediately. 

Once the students completed the step test or were stopped due to the reasons mentioned above, they were told to sit down on the bench and rest. Their pulse rates at 1 and 2 minutes of the rest were then recorded simultaneously with the total duration of the exercise recorded in seconds. 

Heart Rate Recovery in 1 minute (HRR1min) and Heart Rate Recovery in 2 minutes (HRR2min) were next calculated by taking the difference between the highest peak pulse rate during exercise and pulse rate at 1 and 2 minutes rest, respectively. 

### Statistical Analysis

Test for normality was performed for the sample. Analysis was conducted for boys and for girls separately. The participants’ baseline and exercise variables were calculated as mean ±SD and the independent t-test was used as it was appropriate for examining differences for continuous variables with normal distribution. The correlation between each HRR parameters (HRR1min and HRR2min) with body composition measures (BMI z-score, body fat percentage, WC and WHtR) was then determined by using Pearson correlation analysis. Multiple linear regressions were performed separately by sex to determine the strength of association between body composition measurements with each HRR1 min and HRR2 min. For respective boys and girls analysis, there is a separate multiple regression models for each HRR parameters. One consists of HRR1 min as the dependant variable and body composition measurements as independent variables, while the other model consists of HRR2 min as the dependant variable with body composition as independent variables. Age, pulse rate, blood pressure and smoking status were factors that were controlled. All statistical analyses were completed using SPSS version 20 and the level of significance is viewed at p<0.05. 

## Results

A total of 1361 students agreed to participate in the MyHEART study but 285 refused to participate in the exercise test. Two who were acutely ill, two with medical conditions and one with musculoskeletal injuries were excluded. Thus, a total of 1071 students participated in the step test. Their mean age was 12.9 ± 0.3 years (range 12-14) and the average response rate for the study was 51%. The socio-demographic characteristics between respondents and non-respondents in terms of gender and locality were then compared and there was no significant difference shown. 

The characteristics of the participants are shown in Table 1. Boys had a significantly higher WC and WHtR (p<0.001), while for girls it was body fat percentage (p<0.001). There was no significant difference for BMI and BMI z-score in both genders. As for HRR parameters, boys have significantly faster HRR 1 min and HRR 2 min (p<0.001). 

**Table 1:** Baseline characteristics and exercise parameters of the participants.

	**Boys**	**Girls**	**p**
n	405	666	-
Smokers (%)	13.3	2.4	-
Age (years)	12.8±0.3	12.9±0.3	0.028
SBP (mmHg)	110.9 ±10.5	108.6 ±11.9	0.001
DBP (mmHg)	68.9 ±10.6	66.6 ±10.4	0.001
Pulse rate (beats/min)	85.6 ± 14.1	90.0 ±13.2	<0.001
Height (cm)	150.6 ±9.2	150.9 ±6.2	0.455
Weight (kg)	45.7±14.5	45.1±11.5	0.488
BMI (kg/m^2^)	19.9 ±5.4	19.7 ± 4.3	0.353
BMI z-score	0.2 ± 1.6	0.02 ± 1.4	0.060
Body fat (%)	18.9 ± 14.6	25.6 ±10.1	<0.001
WC(cm)	70.6 ± 12.9	67.5 ± 9.8	<0.001
WHtR	0.47 ±0.08	0.45 ±0.06	<0.001
Peak heart rate (beats/min)	177.6 ± 14.5	185.8 ± 11.9	<0.001
Heart rate at 1 min rest (beats/min)	126.8 ± 19.6	144.7 ± 15.5	<0.001
Heart Rate at 2 min rest (beats/min)	115.1 ± 17.8	131.3 ± 14.6	<0.001
HRR 1 min	50.8 ± 13.6	41.0 ± 11.9	<0.001
HRR 2 min	62.6 ± 12.8	54.5 ± 11.9	<0.001

Data are mean ± SD. % = percentage SBP = Systolic blood pressure, DBP = Diastolic blood pressure, HRR = Heart rate recovery, BMI = Body mass index, WC = waist circumference, WHtR = Waist height ratio

The correlation analysis (Table 2) showed that parameters such as BMI z-score, body fat percentage, WC and WHtR were all negatively correlated to HRR 1 min (r = -0.157, -0.189, -0.218, and -0.198 respectively for boys; r = -0.201, -0.214, -0.164, -0.164 respectively for girls). HRR2 min were negatively correlated with all body composition measures in girls (r = -0.107, -0.134, -0.078, and -0.067 respectively), while for boys, only body fat percentage, WC and WHtR (r = -0.101, -0.133, and -0.110 respectively) were negatively correlated with HRR 2 min. 

**Table 2:** Correlation coefficient (r) between HRR parameters and body composition measures using Pearson correlation analysis.

**Body Composition**	**Boys**				**Girls**			
	**HRR 1 min**		**HRR 2 min**		**HRR 1 min**		**HRR 2 min**	
	***r***	***p***	***r***	***p***	***r***	***p***	***r***	***p***
BMI z-score	-0.157	0.001	-0.077	0.061	-0.201	<0.001	-0.107	0.003
Body Fat (%)	-0.189	<0.001	-0.101	0.022	-0.214	<0.001	-0.134	<0.001
WC ( cm)	-0.218	<0.001	-0.133	0.004	-0.164	<0.001	-0.078	0.022
WHtR	-0.198	<0.001	-0.110	0.014	-0.164	<0.001	-0.067	0.043

BMI = Body mass index, HRR = Heart rate recovery, WC = Waist circumference, WHtR = Waist height ratio

The multiple regression analysis (Table 3) showed that only WC was negatively associated with HRR 2 min (p= 0.024) in boys whereas body fat percentage was negatively associated with HRR 2 min (p=0.008) in girls.

**Table 3:** Standardised coefficients (β) between HRR and body composition measures using multiple linear regression after controlling for age, pulse rate, blood pressure and smoking status.

**Body Composition**	**Boys**				**Girls**			
	**HRR 1 min**		**HRR 2 min**		**HRR 1 min**		**HRR 2 min**	
	**β**	**p**	**Β**	**p**	**β**	**p**	**β**	**p**
BMI z-score	0.153	NS	0.132	NS	-0.108	NS	-0.008	NS
Body Fat (%)	-0.102	NS	-0.029	NS	-0.233	NS	-0.325	0.008
WC ( cm)	-0.367	NS	-0.418	0.024	0.192	NS	0.078	NS
WHtR	0.166	NS	0.260	NS	-0.061	NS	0.130	NS

NS = Not significant, BMI = Body mass index, HRR = Heart rate recovery, WC = Waist circumference, WHtR = Waist height ratio

## Discussion

This study showed that there is inverse association of body composition measures with HRR in both boys and girls. For HRR 2 min, WC served as the strongest predictor in boys, while in girls it was body fat percentage.

We compared our study with previous published studies [9-11]. The Children’s Hospital study in Boston revealed BMI for age was associated with HRR 1 min for both genders [9], while The National Health and Nutrition Examination Survey 1999-2002 study showed that WC was the only body composition measures associated with HRR parameters (HRR 1 min, HRR 2 min and HRR 3 min for boys, and HRR 2 min and HRR 3 min for girls)[10]. The European Youth Heart Study, which measured HRR 1 min, HRR 3 min and HRR 5 min, showed that WC was associated with HRR 3 min in boys, but there was no association between body composition measures with HRR parameters in girls[11] . The researchers investigated association between HRR with several cardio-metabolic risk factors which included body composition measures [9-11]. These factors, and the different HRR parameters used, would have led to outcomes that differ from ours. Our study investigated HRR 1 min and HRR 2 min. Both HRR parameters have been validated as an ideal and prognostic measurement for HRR [50].

Another possible reason is the difference in exercise protocols. This study utilised mass screening step test and passive resting recovery while other studies were conducted in controlled laboratory settings, used active resting period for treadmills [9,10] or cycle ergometers [11]. Different exercise protocols have shown to influence HRR outcome [51,52]. 

The difference in results between the genders could be due to variances with fitness level, haematological parameters, and ventricular chamber size [13]. In girls, fat accumulate as total body fat and subcutaneous fat deposits [53]. It is different in boys, as fat accumulation occurs more in the intra-abdominal area, especially visceral adipose tissue [53]. WC has shown to be strongly correlated with boys [25]. These are probable reasons why our result showed that WC was the strongest predictor for HRR in boys and body fat percentage in girls. 

It has been shown that childhood obesity is associated with ANS dysfunction. It has been postulated that obesity is linked to reduced parasympathetic drive [19], but a recent study has revealed that obesity affects both parasympathetic and sympathetic pathways in children [20]. This disparity suggests that more studies are needed in this field.

Since the effect of obesity on ANS control of HRR can occur at an early age, lifestyle changes are important for the well-being of the children. In relation to this, inducing weight loss and introducing lifestyle programmes for overweight and obese children has been shown to improve their HRR [54,55] with the greatest improvement occurring in HRR 1 min [54]. This finding reiterates the importance of introducing a healthy lifestyle to the participants from a young age as it carries many positive effects of exercise and weight control on body composition measures as well as ANS of the heart.

During exercising, the heart rate (HR) increase is due to withdrawal of parasympathetic activity, while further increments in HR are mediated by the sympathetic drive [1,56-58]. During recovery, HRR 1 min is due to vagal reactivation, while HRR 2 min and beyond is due to a combination of vagal drive, reduction in sympathetic pathway, and clearance of metabolites [2,11,12,59]. The increased interest of HRR in adults is its relationship with mortality and it has been shown that decreased vagal activity is a predictor of all-cause mortality in both healthy adults and post myocardial infarction (MI)patients [6-8,60,61]. A study has shown that among MI patients, there is a reduction of parasympathetic drive with dominance of sympathetic pathway [62]. HRR 1 min of less than 12 bpm during active rest [6] or 18 bpm during passive rest [63]indicates higher risk of cardiovascular mortality while HRR 2 min of less than 43 bpm is likewise, also considered unfavourably [7]. 

In children, other than HRR’s association with cardio-metabolic risks, several studies have shown that HRR may be considered as a marker for CVS health [13,64], and for the assessment of parasympathetic tone in post congenital cardiac surgery [65,66]. 

The strength of this study lies in the large sample of participants. The modified Harvard Step Test which was used as a tool in the study population suggests that it could be considered as an alternative tool for fitness assessment. The step test is relatively safe, simple, cheap, and portable[67]. In this study, five students could perform the exercise simultaneously since it can be conducted within a small space, and it requires minimal equipment. Moreover, not much specialized training is required and since it is not a laboratory setting, the atmosphere is more relaxed. The time taken for conducting each test was usually less than 10 minutes and this short duration is ideal for on field mass testing. Within a period of three months, the study was able to cover 15 schools within the three zones in Malaysia. Further studies are warranted to compare the modified Harvard Step Test with other established fitness assessment protocols.

As is present in all studies, there are limitations. Since the sample is of the same age group, the results reflected in this study may not exhibit the abilities of the rest of the paediatric population. Moreover, the cross sectional design used in this study does not establish causality. A longitudinal study on young adults has indicated that obesity and cardio-metabolic risks develop first prior to any effects on HRR[68], and since MyHEART is an on-going longitudinal study, it is our plan to investigate this matter on the current group of adolescents. 

## Conclusion

This study has shown that body composition measures are inversely associated with HRR in healthy Malaysian adolescent with waist circumference as the strongest predictor for boys and body fat percentage for girls respectively. MyHEART is a continuing longitudinal study, with future plans for follow-up at 15 years old (MyHEART II, 2014) and 17 years old (MyHEART III, 2016). This would enable us to determine how lifestyle factors operating in early adolescence affect cardio-metabolic health in early adulthood, and to assist the design and development of effective prevention program in Malaysia. It is also our aim to investigate the longitudinal association between HRR with body composition measures. 
